# Re-emerging of Marburg virus: warning about its virulence and potential impact on world’s health

**DOI:** 10.1097/JS9.0000000000000162

**Published:** 2023-01-18

**Authors:** Shopnil Akash, Talha B. Emran, Hitesh Chopra, Kuldeep Dhama

**Affiliations:** a Department of Pharmacy, Faculty of Allied Health Sciences, Daffodil International University, Dhaka; b Department of Pharmacy, BGC Trust University Bangladesh, Chittagong, Bangladesh; c Chitkara College of Pharmacy, Chitkara University, Rajpura, Punjab; d Division of Pathology, ICAR-Indian Veterinary Research Institute, Bareilly, Izatnagar, Uttar Pradesh, India

Highlights

- Last couple of years, numerous viral infections are making life-threatening.
- MVD is a highly life-threatening hemorrhagic fever.
- Marburg virus is categorized under *Mononegaviruses* within the family *Filoviridae*.
- Marburg viruses may enter the body via fractures or wounds in the skin.

*Dear Editor*,

Over the last couple of years, numerous viral infections are making life-threatening and problematic conditions around the globe, and continuously many people have died. These infectious illnesses include severe acute respiratory syndrome coronavirus 2 (SARS-CoV-2)^1^, *Langya henipavirus* 2, and monkeypox^3^, among others. At the same time, another pathogenic epidemic is likely to harm global health systems, which cause by Marburg viral disease (MVD). MVD is a highly life-threatening hemorrhagic fever that may infect humans and nonhuman creatures. According to the findings, it has been linked with high morbidity and fatality rates ranging from 24 to 90% due to MVD^4^.

Marburg virus (MARV) was initially recorded in 1967 in Marburg, Germany, when researchers conducted studies on tissue taken from African green monkeys imported from Uganda to develop polio vaccines. Plasma from affected guinea pigs was investigated using electron microscopy to determine and characterize the virus, and then it was given the name ‘Marburg virus.’ Multiple simultaneous outbreaks were reported in both Yugoslavia and Frankfurt. In 1975, another MVD epidemic in South Africa affected three persons. Human-to-human transmission allowed the first infected person to transmit to his traveling partner and a nurse in Zimbabwe. In 1980, a third MVD epidemic was revealed in Kenya; a man contracted the disease after seeing Kitum Cave, and a doctor acquired it while helping him. In 1987, a new strain of MARV was discovered after a short outbreak in Kenya. Nevertheless, a 15-year-old Danish kid became infected 7 days afterward, visiting Kitum cave^5^,^6^.

MARV is categorized under *Mononegaviruses* within the family Filoviridae and belongs to the genus *Marburgvirus*. The components of a virion are typically cylindrical and filamentous. However, rod-shaped, ring-shaped, and U-shaped variants may occur. The virion has a diameter of around 80 nm and a length of 790 nm but varies greatly. A negative-strand RNA genome of ∼19 kbp in size is protected inside a helical nucleocapsid. Glycoprotein is one of seven structural proteins encoded by the genome and serves a crucial function in facilitating viral entrance into host cells. Virions have glycoprotein spikes all over them, extending outward from the particle surface by a distance of 5–10 nm^7^,^8^. MARVs may enter the body via fractures or wounds in the skin, and the liver, lymph nodes, and spleen are the initial sites of infection; the virus swiftly spreads to other tissues in the body. MARVs primarily attack cells of the immune system, particularly monocytes and dendritic cells. As a result, they inhibit the activation of the immune system and make it possible for the virus to replicate uncontrollably. Even if the virus does not immediately contaminate lymphocytes, considerable percentages of these cells nonetheless experience apoptosis. This outcome is a characteristic of the pathogenesis of MVD^8^,^9^.

Physical interaction with contaminated blood, secretions, or body fluids (via damaged skin or mucous membranes) may cause circulation from one individual to another. Objects and fabrics (such as clothing and bedding) infected with these fluids and secretions may also serve as vectors for the spread of MVD. Since the virus has been found in the sperm of a man affected, it is reasonable to assume that sexual transmission is also possible. Male sufferers of MVD are encouraged to delay returning to sexual activity until at least 12 months, during which time they should engage in safer sex practices. They are the primary carriers of disease^10^.

The disease has three stages: the initial spread, when certain organs are affected, and the recovery phase when those organs are late organ/healing phase. In humans, the incubation time is 5–9 days on average but may be as long as 21 days. Fever, headache, vomiting, diarrhea, and extreme myalgia are just some of the vague first symptoms that may be present. A typical sign is muscle soreness or discomfort. It is possible to have severe diarrhea with a lot of water, cramps in the abdomen, and vomiting during the first week of sickness^10^,^11^. Malaria, typhoid, meningitis, and other viral hemorrhagic fevers share many nonspecific symptoms, making a correct diagnosis more difficult.

Diagnosing MVD clinically might be difficult at times. However, swabs or blood should be run via a reverse-transcriptase PCR to check for MVD. If the test is positive, the MVD has been established. A negative effect does not rule out illness until at least 72 hours of symptoms have been observed^11^,^12^. Additional methods for detecting MVD should be available, such as the enzyme-linked immunosorbent test for antibodies and the cell culture-based viral isolation method^13^.

At this time, neither a vaccine nor an antiviral medicine has been approved for the treatment of MVD. The provision of supportive therapy, which may include rehydrating the patient with fluids either orally or intravenously and addressing specific symptoms, may enhance the likelihood of the patient surviving their condition^14^. Only a limited amount of research has been published on what is now being developed to combat MARV. These antiviral nucleoside analogs, phosphorodiamidite, immunotherapeutic, and morpholino oligomers are some of them. It would seem that favipiravir and remdesivir might be beneficial in nonhuman primates; however, there is no evidence to suggest that they may be acceptable treatments in humans at this time. It has been established that galidesivir is effective against MARV for a period of up to 24 hours^15^. Due to the high probability of spreading, MARV is classified as a category-4 illness. It is, therefore, of the utmost importance to rapidly advance scientific research and development of effective medications for treating MVD. Health professionals, legislators, and government officials will need to collaborate to create effective safety and preventive regulations to prevent the virus from spreading from Africa to other countries. and control the disease outbreak that’s happening right now.

## Provenance and peer review

Not commissioned, internally peer-reviewed.

## Ethical approval

Not applicable.

## Sources of funding

None.

## Authors’ contributions

S.A.: conceptualization, data curation, writing – original draft preparation, writing – reviewing and editing. T.B.E.: writing – reviewing and editing, visualization, supervision. H.C. and K.D.: data curation, writing – original draft preparation, writing – reviewing and editing.

## Conflicts of interest disclosure

The authors declare that they have no financial conflict of interest with regard to the content of this report.

## Research registration unique identifying number (UIN)

Not applicable.

## Guarantor

Talha B. Emran.
